# Prevalence of Internet addiction and its associated factors among female students at Jouf University, Saudi Arabia

**DOI:** 10.1186/s42506-019-0009-6

**Published:** 2019-02-26

**Authors:** Doaa M. Abdel-Salam, Hajar I. Alrowaili, Haifa K. Albedaiwi, Amnah I. Alessa, Hanan A. Alfayyadh

**Affiliations:** 1 Department of Family and Community Medicine, College of Medicine, Jouf University, Sakakah, Saudi Arabia; 2 Department of Public Health and Community Medicine, Faculty of Medicine, Assiut University, Asyut, Egypt; 3 Medical student College of Medicine, Jouf University, Sakakah, Saudi Arabia

**Keywords:** Internet addiction, Prevalence, University students

## Abstract

**Background and objectives:**

Internet addiction is an increasing problem among university students worldwide. The Internet provides numerous educational advantages, but too much Internet use can lead to unfavorable outcomes such as social isolation and poor academic achievement. The objectives of the present study were to assess the prevalence of Internet addiction and its associated factors among female students at Jouf University, Saudi Arabia.

**Methods:**

A cross-sectional study using a multistage proportionate sampling technique was done. A self-administered questionnaire was distributed to the female students of Jouf University during face-to-face interviews with them. This questionnaire consisted of two parts; the first is a structured one for identifying sociodemographic features, and the second is Young’s Internet Addiction Test (YIAT) to assess Internet use among the students.

**Results:**

According to the YIAT scale of Internet addiction, 48.6% of the students were scored to be average Internet users. However, 49.5% and 1.9% of the students had moderate and severe addictions, respectively. The vast majority of students (94.6%) preferred home to access the Internet. Communication was the main purpose of using the Internet as it was reported by 47.3% of the students. The majority of the students (79.5%) utilized mobile phones for Internet access while other devices such as a laptop, tablets and desktop were used by 15.4%, 4.3%, and 3.2% of the students, respectively. More than half of the students (54.6%) used the Internet for an average of more than 4 h every day. Also, more than half (51.4%) used it in the evening being the dominant time of using the Internet. Internet addiction was significantly higher among students with high father education, students who sleep 6 h or less, students who utilize the Internet for entertainment purposes, and students who utilize the Internet mainly in the midnight.

**Conclusion:**

Nearly half of female students at Jouf University have moderate or severe Internet addiction. The significant predictors of Internet addiction were sleeping hours ≤ 6, midnight as the dominant time for utilizing the Internet and using the Internet for entertainment purposes.

## Introduction

The Internet is considered to be the most widely used media in the world, and it varies from other types of media. The causes of this widespread accessibility are the Internet has numerous activities that attract its users, the Internet displays a chance to communicate with people all over the world without any restriction, and young adults have become an important goal of this commercial concern [1]. There has been a tremendous growth of Internet use all over the world, and this is anticipated to continue with its use, becoming an essential part of daily life. Completing work, playing games online, reading and writing emails, and engaging in communication are common activities involving Internet use [2]. Internet activities and technologies that are increasing rapidly have attracted young adults, leading to excessive use of the Internet and maladaptive Internet attitude known as “Internet addiction” [3]. The term “addiction”, even traditionally utilized to describe a physical dependence of substances, has been applied to the excessive use of the Internet [4]. Internet addiction disorder is expressed as too much computer use that contradicts daily activities and can harm daily function [5].

Various studies showed that adolescents are the most exposed group to Internet addiction as they carry communication with others on social network sites rather than the actual contact in real life [6]. University students are thought to be at a hazardous risk to excessive Internet use worldwide. Internet addiction among these students was established to be associated with different psychiatric disorders such as depression, stress, anxiety [7], low self-respect [8], and low psychosomatic well-being [9].

International estimates of Internet addiction mostly differ. In a large European study (2014), the prevalence of Internet addiction among adults was between 7.9% and 22.8% [10]. Another study in Europe (2012) reported a prevalence of Internet addiction of 4.4% among young adults. [11]. Among Korean adolescents, 1.6% have reported to experience Internet addiction and 38% were liable to Internet addiction [12]. The prevalence of Internet addiction has been shown to be up to 26% in some studies [13–15].

Internet addiction prevalence was 2.6% among adolescents in El-Minia, Upper Egypt, while the prevalence of potential Internet addiction was 18.2% [16]. Another study conducted among adolescents recruited from private and governmental schools in Cairo, Egypt, revealed a prevalence of 0.8% [17]. Saudis are heavier Internet users than others in different countries. A study was done among Taif University students in Saudi Arabia and showed that most of them (98.2%) were using the Internet. Internet addiction was reported among 4% of them whereas potential Internet addiction was among 45.3%. The Internet users in this study used to access the Internet predominantly from home, followed by college, and then Internet cafes. The average time of using the Internet was more than 5 h per day among 40% of the students [15].

Many tools have been used to screen for Internet addiction. The first tool was the Internet Addiction Test (IAT) published by Young in 1998. IAT is a 20-item scale that rates the grade of preoccupation, compulsive use, behavioral abnormalities, emotional problems, and effect on general functioning associated with Internet use [3].

Internet addiction is an increasing problem among university students all over the world. In addition, no study about Internet addiction could be traced among university students in the Jouf region in Saudi Arabia. The present study was intended to determine the prevalence of Internet addiction among female students at Jouf University in Saudi Arabia and to identify its associated factors.

## Participants and methods

### Setting

The Kingdom of Saudi Arabia (KSA) has a population of 32.6 million people. It constitutes one of the countries that have demographic transition in its population structure. Jouf region is located in the northern part of KSA. Jouf University is located in the Jouf region and is currently the only university serving the region. The female campus in Jouf University includes eight colleges: Medicine, Pharmacy, Computer Science, Science, Applied Medical Sciences, Administrative Sciences, Education, and Sharia. The total number of female students registered in Jouf University according to records of the academic year 2016–2017 was 7040.

### Study design and sampling

The present study was a descriptive cross-sectional study conducted among female students of Jouf University during the academic year 2016–2017. A self-administered questionnaire was distributed to the female students of the University during face-to-face interviews with them.

The sample size was calculated assuming the prevalence of Internet addiction is 4% [15] with a precision of 5%, applying a confidence level of 95% and 80% power of the study. The calculated sample size was 368 students.

A multistage proportionate sampling technique was used. A sampling fraction was calculated to choose participants in relation to the number of students in each college. For each college, students were selected using a systematic random sampling method (using the academic identification number) from the available records. The timing of the study was selected to avoid the start and end of the semester, when students were absent, settling into a routine, or preparing for final exams.

### Inclusion criteria

Active Internet user since at least 1 year

Willing to give consent and complete the questionnaire

### Exclusion criteria

Not willing to give consent and complete the questionnaire

Not using the Internet

### Instrument

A self-administered anonymous questionnaire was applied. It consisted of two parts. The first part included socio-demographic features such as age type of college, educational level of parents, residence, marital status, number of sleeping hours, and family income.

The second part of the questionnaire was the Arabic version of Young’s Internet Addiction Test (YIAT) that was validated in a preceding study conducted in Lebanon [18]. YIAT is the first valid and reliable tool for Internet addiction. This 20- item questionnaire was developed by Kimberley Young. It classifies Internet addiction into mild, moderate, and severe degrees. Each answer is scored on a Likert scale from 1 to 5: score 1 = rarely, 2 = occasionally, 3 = frequently, 4 = often, and 5 = always. The final score is obtained by summing up the scores of all questions. The greater score represents a higher level of addiction. Scores between 20 and 49 mean normal Internet use, 50–79 mean moderate Internet use (moderate addiction), and 80–100 mean severe Internet use (severe addiction) [2].

### Statistical analysis

Data analysis was done using SPSS version 16 (SPSS Inc., Chicago, USA). Descriptive statistics and chi-square test were performed. Factors significantly affecting the prevalence of Internet addiction on univariate analysis were entered into a multivariate logistic regression analysis. A level of *p* < 0.05 was considered statistically significant.

## Results

Table 1 shows the socio-demographic characteristics of the respondents. The mean age of the studied students was 20.85 ± 1.73 (18–26 years old). Nearly 65% of the respondents were from scientific colleges and 35% from theoretical colleges. Most of the students were single (82.2%). A considerable proportion of their fathers and of their mothers had attained university education or above (44.3% and 41.8%, respectively). The majority of the students (92.4%) lived with their families and the minority (7.6%) lived in university housing. The number of sleeping hours per day was 7–8 among 41.9% of the students. The monthly family income exceeded 10,000 RS among 49.7% of the students.

**Table 1:** Sociodemographic features of female students at Jouf University, SA, 2017

	No. (*n* = 370)	Percent
Age (years)		
≤ 20 years	170	45.9
> 20 years	200	54.1
Mean ± SD	20.85 ± 1.73 (18.0–26.0)	
Type of college		
Scientific	242	65.4
Theoretical	128	34.6
Marital status		
Single	304	82.2
Married	66	17.8
Father education		
Illiterate	18	4.9
Read and write	53	14.3
Primary	24	6.5
Preparatory	17	4.6
Secondary	94	25.4
University	132	35.7
Postgraduate	32	8.6
Mother education		
Illiterate	38	10.3
Read and write	54	14.6
Primary	38	10.3
Preparatory	24	6.5
Secondary	61	16.5
University	125	33.7
Postgraduate	30	8.1
Living arrangement		
Living with family	342	92.4
University housing	28	7.6
No. of sleeping hours		
6 h or less	142	38.4
7–8 h	155	41.9
9 h or more	73	19.7
Family income		
< 5000 RS	77	20.8
5000–10,000 RS	109	29.5
10,001–15,000 RS	77	20.8
> 15,000 RS	107	28.9

**Fig. 1:**
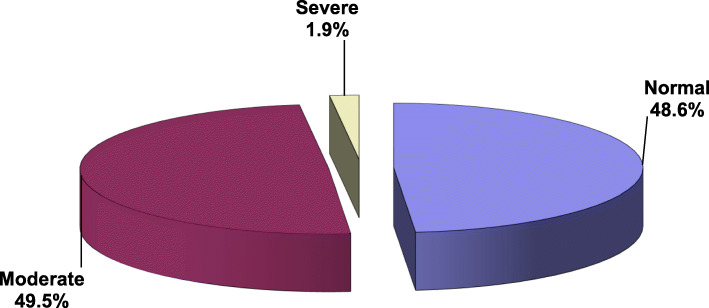
Degree of Internet addiction among the respondents

Figure 1 describes the pattern of Internet use among the students. According to the YIAT scale of Internet addiction, 48.6% of the students are scored normal Internet users. However, 49.5% and 1.9% of the students had moderate and severe addictions, respectively. Table 2 the vast majority of the students (94.6%) preferred home to access the Internet. Communication was the main purpose of using the Internet (47.3%). The majority of the students (79.5%) utilized mobile phones for Internet access while other devices such as laptop, tablets, and desktop were used by 15.4%, 4.3%, and 3.2% of the students, respectively. More than half of the students (54.6%) used the Internet for an average of more than 4 h daily. Also, more than half (51.4%) used the Internet in the evening.

**Table 2:** Pattern of Internet use of female students at Jouf University, SA, 2017

	No. (*n* = 370)	%
Favorite places for using the Internet^#^		
Home	350	94.6
Cafe	127	34.3
University	112	30.3
Main purposes of using the Internet^#^		
Communications	175	47.3
Information	92	24.9
Entertainment	75	20.3
Others	56	15.1
Devices utilized for Internet access^#^		
Mobile phones	294	79.5
Laptop	57	15.4
Tablets	16	4.3
Desktop	12	3.2
Average duration of utilizing the Internet		
< 2 h	43	11.6
2–4 h	125	33.8
> 4 h	202	54.6
Dominant times for utilizing the Internet		
Morning	32	8.6
Afternoon	24	6.5
Evening	190	51.4
Midnight	124	33.5

^#^More than one answer had been reported

Table 3 showed a statistically significant relationship between Internet addiction and number of sleeping hours (*p* < 0.0100). Nearly 50% of students with moderate or severe Internet addiction reported sleeping for 6 h or less compared to 25.6% of students with normal Internet use. The prevalence of Internet addiction was significantly higher among students with high father education (*p* < 0.0100). However, no statistically significant differences were found between age, type of college, marital status, mother education, living arrangement, and family income on one hand and Internet addiction on the other hand.

**Table 3:** Relationship between Internet addiction and sociodemographic features of female students at Jouf University, SA, 2017

	Degree of Internet addiction				*p* value
	Normal (*n* = 180)		Moderate/severe (*n* = 190)		
	No.	Percent	No.	Percent	
Age (years)					0.440
≤ 20 years	79	43.9	91	47.9	
> 20 years	101	56.1	99	52.1	
Type of college					0.071
Scientific	126	70.0	116	61.1	
Theoretical	54	30.0	74	38.9	
Marital status					0.809
Single	147	81.7	157	82.6	
Married	33	18.3	33	17.4	
Father education					
Illiterate/read and write	51	28.3	20	10.5	0.000*
Basic education	22	12.2	19	10.0	
Secondary	46	25.6	48	25.3	
University or more	61	33.9	103	54.2	
Mother education					0.917
Illiterate/read and write	45	25.0	47	24.7	
Basic education	28	15.6	34	17.9	
Secondary	29	16.1	32	16.8	
University or more	78	43.3	77	40.5	
Living arrangement					0.069
Living with family	171	95.0	171	90.0	
University housing	9	5.0	19	10.0	
No. of sleeping hours					0.000*
6 h or less	46	25.6	96	50.5	
7–8 h	90	50.0	65	34.2	
9 h or more	44	24.4	29	15.3	
Family income					0.376
< 5000 RS	35	19.4	42	22.1	
5000–10,000 RS	56	31.1	53	27.9	
10,001–15,000 RS	32	17.8	45	23.7	
> 15,000 RS	57	31.7	50	26.3	

*p* value was calculated using the chi-square test

^*^Significant at *p* <0.05

The relationship between Internet addiction and the pattern of Internet use is depicted in Table 4. A statistically significant relationship was found between Internet addiction and main purposes of using the Internet as addiction was significantly higher among those using internet for entertainment purposes (*p* < 0.0100). Also, internet addiction was significantly higher among students using internet in the midnight however, no statistically significant differences were found between favorite places for using the Internet, devices utilized for Internet access, and average duration of utilizing the Internet on one hand and Internet addiction on the other hand.

**Table 4:** Relationship between Internet addiction and the patterns of Internet use of female students at Jouf University, SA, 2017

	Degree of Internet addiction				*p* value
	Normal (*n* = 180)		Moderate/severe (*n* = 190)		
	No.	Percent	No.	Percent	
Favorite places for using the Internet					0.815
Home	174	96.7	176	92.6	
Cafe	59	32.8	68	35.8	
University	54	30.0	58	30.5	
Main purposes of using the Internet					0.000*
Communications	88	48.9	87	45.8	
Information	60	33.3	32	16.8	
Entertainment	25	13.9	50	26.3	
Others	24	13.3	32	16.8	
Devices utilized for Internet access					0.308
Desktop	3	1.7	9	4.7	
Laptop	31	17.2	26	13.7	
Tablets	7	3.9	9	4.7	
Mobile phones	144	80	150	78.9	
Average duration of utilizing the Internet					0.418
< 2 h	23	12.8	20	10.5	
2–4 h	65	36.1	60	31.6	
> 4 h	92	51.1	110	57.9	
Dominant times for utilizing the Internet					0.003*
Morning	14	7.8	18	9.5	
Afternoon	17	9.4	7	3.7	
Evening	103	57.2	87	45.8	
Midnight	46	25.6	78	41.1	

*p* value was calculated using the chi-square test

^*^Significant at *p* <0.05

In logistic regression analysis (Table 5), the significant factors associated with moderate or severe Internet addiction were sleeping hours ≤ 6, midnight as the dominant time for utilizing the Internet and using the Internet for entertainment purposes.

**Table 5:** Logistic regression analysis of risk factors of moderate/severe Internet addiction

	*p* value	OR	95% CI	
			Lower	Upper
No. of sleeping hours				
6 h or less	0.018	2.6	1.2	6.1
Dominant time for utilizing the Internet				
Midnight	0.029	3.5	2.3	9.5
Entertainment is the main purpose for using the Internet	0.021	3.3	1.4	10.4

*OR* odds ratio, *CI* confidence interval

## Discussion

A dramatic difference in Internet use had occurred in the late 1990s and early 2000s. It represents the revolution in communication and information technologies. The most probable effect of this was the huge growth of Internet use all over the world [4]. The prevalence of Internet use is increasing in Saudi Arabia. The fast growth of Internet use has been associated with queries regarding its effect, both positive and negative on the society and users [15]. The present study showed that severe and moderate Internet addictions were reported among 1.9% and 49.5% of the students, respectively, whereas the remaining 48.6% of the students were considered to be non-addicts. The high prevalence of Internet addiction revealed in the present study was justified by Young, 2004, who said that university students have much unstructured time. They always look for doing communication through the Internet and use the Internet to leave the university sources of stress from exams and studying [3]. A study conducted by Ismail among adolescents in Zagazig, Egypt, 2007, revealed that the overall prevalence of Internet addiction was 54.6% [14]. A study done among secondary school students in Riyadh city in Saudi Arabia, 2013, revealed that the prevalence of severe Internet addiction was 5.3% [19]. Also, a study conducted by Alshehri and his colleagues found that the prevalence of moderate and severe Internet addictions were 45.3% and 4%, respectively, among Taif University students in Saudi Arabia [15]. Severe Internet addiction was found among 13% of Menoufia University students in Egypt, 2015 [20]. The observed differences in the prevalence of Internet addiction in the aforementioned studies even in studies conducted in the same country could be attributed to the application of various assessment instruments, cutoffs, and the differences in the cultural and social contexts.

This study revealed that the majority of the students had home Internet access especially through their mobile phones, and this was consistent with other studies [17, 21]. A long time spent on the Internet reaching more than 4 h per day is also a character of Internet addiction in the present study. This was also a finding of other studies [15, 22]. This is due to the inability of the students to restrict their Internet use particularly when they involve in communication websites and the availability of 24-h Internet access at their homes. The present study showed that students with moderate and severe addiction mostly used the Internet at midnight. This is consistent with other studies [19, 23]. The use of the Internet in the night and midnight leads to academic, social, or occupational problems which even might exacerbate Internet addiction among these university students [24].

More than 50% (54.2%) of students with moderate and severe Internet addiction had a highly educated father compared to students with normal Internet use. This is consistent with other studies [20, 25]. This is because the tendency of highly educated parents to socialize their children into the world of modern information technology [26].

In the present study, students with moderate and severe addiction mostly used the Internet mainly for entertainment purposes while non-addicts used it mainly for information purposes. This finding is consistent with other studies [15, 27]. Young found that non-addicts mainly used the Internet in the aspects of gathering information. However, addicts or potentially addicts predominantly used it for entertainment purposes [3]. Other studies [20, 28, 29] showed that students with severe addiction used the Internet for alleviating the sense of loneliness and entertainment purposes rather than other purposes.

Internet addiction was significantly associated with short sleeping time (6 h or less) in the present study. Young noticed the association between short sleep and Internet addiction. He proposed that sleep pattern is disrupted due to Internet use in late night. This deficient sleep leads to excessive fatigue often resulting in functional or academic impairment and decreases one’s immune system leaving persons susceptible to diseases [3].

### Limitations of the study

The administrative authorities in Saudi Arabia prohibit female researchers from performing studies on male students. So, the researcher did not have the opportunity to estimate sex-specific prevalence rates. Another limitation is being a cross-sectional study which revealed the relation between Internet addiction and some risk factors without being able to determine a cause-effect relationship.

## Conclusion and recommendations

The present study provided valuable information about Internet addiction among female students at Jouf University. Although the present study revealed that the prevalence of Internet addiction was not higher than other universities and populations, the reported rates of Internet addiction denote a rising problem worldwide. Thus, concentrating on related factors can help in implementing more effective intervention programs for the vulnerable group of Internet addiction.
